# Supplementary material for: Patterns of Intron Gain and Loss in Fungi
Source: PLoS Biol. 2004 Nov 30;2(12):e422. doi: 10.1371/journal.pbio.0020422 (PMC532390; doi:10.1371/journal.pbio.0020422)
Supplement: Table S1 — Also available at http://genes.mit.edu/NielsenEtAl/. (4.3 MB ZIP). [file pbio.0020422.st001.zip › NielsenEtAl/html/748.html]

AN6709.1.NCU07658.1.MG09877.1.FG04168.1


```
 CLUSTAL W (1.82) Multiple Sequence Alignments - Introns Inserted


Sequence 1: MG09877.1	2745 aa
Sequence 2: FG04168.1	1814 aa
Sequence 3: NCU07658.1	1940 aa
Sequence 4: AN6709.1	1999 aa
Alignment Length: 2960 aa
Number Identitical Residues: 847 aa
Alignment Score (without introns) 42737


MG09877.1 	------------------------------------------------------------
NCU07658.1	------------------------------------------------------------
FG04168.1 	------------------------------------------------------------
AN6709.1  	MAEAENDPTNELNQTSVTQADKQNGVDVATEPHAPEVVAESKPALTDESERQEIPTIKEN
          	 :.:....:.. ..:: :.:..... . ::.. :..  :.:..: :..:. .. .: ...

MG09877.1 	------------------------------------------------------------
NCU07658.1	------------------------------------------------------------
FG04168.1 	------------------------------------------------------------
AN6709.1  	EDTMANNRLNDSKNNLPHEPSVTSPDTTTDSNEPTDEPEQPHTEGDQLETLQQDQPPASD
          	..: :..  ..:... . ..: ::..:::.:...:...... :.... .: ......::.

MG09877.1 	-----------------------------------------MASLQFVVSSLETIAK--D
NCU07658.1	-----------------------------------------MSSLKFVTSSLDVIAA--N
FG04168.1 	-----------------------------------------MSSLKFVVSSLEAIAASKD
AN6709.1  	EQLNEAPDAPSTRDEQLAQDMRQRSDSRSTTATFATNRSSVVSSTVFIVTALDAIGASRE
          	.. ..:..:.:: ... :..  . :.: ::::: ::. :: ::*  *:.::*:.*. : :

MG09877.1 	SHRNKQLSESVQTALAAIKESDPQLPDPEVIFAPLQLATKSGSVPLITNALDSIGKLTSS
NCU07658.1	AGKNKELAELAQKAQEAIKTNDQQLPDPEVIFAPLRLATKSGTIPLTTTALDCIGKLISS
FG04168.1 	AQRNKQLEETTTKALDAIKEQD-QLPDPEIVFAPLQLASRSTNVQLTTTALDCIGKLISY
AN6709.1  	ARKSKELEDAVKNALANVKQSDRQPIDPEILFYPLLLASRTLSIPLQVTALDCIGKLITY
          	: :.*:* : . .*   :* .* *  ***::* ** **::: .: * ..***.**** : 

MG09877.1 	SYFSQLS-----SQEGS----AERAPLIERAIDTICDCFQGETTHADVQLQIVKSLLAAV
NCU07658.1	TYFSVPSGRSAAASEDGQPQQQQYLPLIERAIDTICDCFQGEATLVEIQLQIVKSLLAAV
FG04168.1 	SYFSVPSNPSEGTEEGA----EPVAPLIERAIDTICNCFQGETTAVEIQLQIVKSLLAAV
AN6709.1  	SYFAFPSAQEAKPSEADAT--AEQPPLIERAIDAICDCFENEATPIEIQQQIIKSLLAAV
          	:**:  *  .  ..*   .      ********:**:**:.*:*  ::* **:*******

MG09877.1 	LNDKIIVHGAGLLKAVRQVYNVFLLSRSPVNQQVAQGTLTQMAGTVFERVKTRLHMKESR
NCU07658.1	LNDKIVVHGAGLLKAVRQVYNIFLLSRSTANQQVAQGTLTQMVGTVFERVSARLHMREAR
FG04168.1 	LNDKIVVHGAGLLKAVRQVYNVFLLSRSTANQQVAQGTLTQMVGTVFERVKTRLHMKEAR
AN6709.1  	LNDKIVVHGAGLLKAVRQIYNMFIYSKSSQNQQIAQGSLTQMVSTVFDRLRVRLDLRELR
          	*****:************:**:*: *:*. ***:***:****..***:*: .**.::* *

MG09877.1 	LNLSRLAQSSSKLTLEVQESINGSSAPSADYDDRDSDAGGETPVERRD-----EDAKLTI
NCU07658.1	ANLSKLKRSQSSFNVNGSSDGQNSTQEEANGDDDENVPEDASESQLQDGDGNGDGPKLTL
FG04168.1 	LNLEHLKNGSSNATFDHAESTNGAND-NGDRDESPAEPSEAADSEPAE-----SGAKLTL
AN6709.1  	IREGEKAQAGSSESVTIEPVVSPPSAEDDQASDVASVAADQPVSKEPT-------EKLTL
          	 .  .  .. *. ..      . ..  . : .:    .   .  :           ***:

MG09877.1 	KDLEKMKNFDDSGLGDGPTMVSQVKPAKRVARSISENTQG-DASLDDSPEALDAEDEVYI
NCU07658.1	KDLEHRKSFDDSNLGDGPTMVTQLKPNRKPARSVSEQSVP-ESAQEDSPESLDAEDEVYI
FG04168.1 	KDLEHRKSFDDSNLGDGPTMVTRLKPERKETGTPASDQAGQDSTPAEDGDVLDAEDEVYI
AN6709.1  	ESFESNKDVTTVN-DNVPTMVTRANINQKRTQSYSGTSSE-EKEAEDASSNEDDVDEIYV
          	:.:*  *..   . .: ****:: :  :: : : :      :    :  .  *  **:*:

MG09877.1 	RDAYLVFRSFCNLSTKVLSQDQLYDLRGQAMRSKLISLHLIHTLLHSNIAVFVSPLCTIT
NCU07658.1	RDAYLVFRSFCNLSTKILPPDQLYDLRGQPMRSKLISLHLIHTLLNNNISVFTSPYCTIT
FG04168.1 	RDAYLVFRSFCNLSTKVLPPDQLYDVRGQPMRSKLISLHLIHTLLNNNIAVFTSPFCTIK
AN6709.1  	KDAFLVFRALCKLSHKVLSHEQQQDLKSQNMRSKLLSLHLIHYLINNHVIIFTTPLLTLK
          	:**:****::*:** *:*. :*  *::.* *****:****** *::.:: :*.:*  *:.

MG09877.1 	NTK-NNEPTSFLEAIKYYLCLSITRNGASSVDKVFD~VTCEVFWLILKFMRASFK0KEIE
NCU07658.1	NTK-NNEPTSFLQATKYYLCLSITRNGASSVDRVFN~ICCEIFWLMLKYMRAPFK0KEIE
FG04168.1 	NSK-SGDPTSFLQAIKYYLCLSITRNGASSVDRIFN0------------------~KEIE
AN6709.1  	NSSGNLEAMTFLQAIRPHLCLSLSRNGASSVPKVFE~VCCEIFWLMLKHMRVMMK0KELE
          	*:... :. :**:* : :****::******* ::*:   ..      .      . **:*

MG09877.1 	VVLNEIYLALLARKTAPLSQKLYFVGILNRLCADPRALVEVYLNYDCDRN-VDNIFQT~L
NCU07658.1	VFLNEIYLALLARRTAPLSQKLAFVSILKRLCEDPRALVEFYLNYDCDRN-VDNIFQR~I
FG04168.1 	VFLNEIYLALLARRTAPLSQKVQFITILNRLCADPKALVEIYLNYDCDQT-VDNIYQT~I
AN6709.1  	VFMKEIYLAILEKRNAPAFQKQYFMEILERLADEPRALVEMYLNYDCDRTALENIFQN2I
          	*.::*****:* ::.**  **  *: **:**. :*:****.*******:.:::**:*  :

MG09877.1 	IEDLSRFATAPVAITPAQEQLFEERHSKGNQAT-DWQTKGAMPPPLSATQIAPQNEI-ES
NCU07658.1	VEDLSKFATSSTTVTALQEQQYEENHAKYGSAS-EWQMRNVLPPPLTVALIAQNTDT-DG
FG04168.1 	IEDLSKFSTTPLTITTINEQVYEEMRLKTTPAS-EWQLKTTLPPPLTVAHIAPHQDS-EP
AN6709.1  	IEQLSRYASIPTVVNPLQQQQYHELHVKASSVGNEWHQRGTLPPNLTSASIGNNQQPPTH
          	:*:**:::: . .:.. ::* :.* : *   . .:*: : .:** *: : *. : : .  

MG09877.1 	DIPKEYAIKRMSLDSLVEALRSLVNWSQSGRPDGNAGTVNESERRSSLEDARDSIDPSYS
NCU07658.1	DIPKDYVLKRQALDSLVESLRSLLNWSHPGRPEVITTGTGISERRPSSDEIRESMDPSIM
FG04168.1 	DYPKEYAIKRLSIEALVETLRSMVNWSAPIRGDAEP-THPENQDIKGSLDIRPSIDPSIN
AN6709.1  	SVPSEYILKHQAVECLVVILESLDNWASQRSVDPTAARTFSQKSVDNPRDSMDSSAPAFL
          	. *.:* :*: :::.**  *.*: **:     :  .     .:   .  :   *  *:  

MG09877.1 	--EGLSRGDTPALPSTPVIDDDPEHLEKEKARKTAMTNAIKVFNFKPKKGIALLLKEGFI
NCU07658.1	G-ESLSRLDTPTMPSTPLLDDDPDQLEKEKQRKTALGNAIRVFNYKPKNGIKLLLKEGFI
FG04168.1 	--DSVSRVETPLPPSTPILEDDPDQLEKEKMRKTALMKGINQFNFKPKKGIQMLIRDGFI
AN6709.1  	ASPRVDGADGSTGRSTPVPDDDPSQVEKVKQRKIALTNVIQQFNFKPKRGVKLALQEGFI
          	.:  :.  : .   ***: :***.::** * ** *: : *. **:***.*: : :::***

MG09877.1 	ASDRPEDIAKFLIQEERLDKAQIGEYLGEGEPKNIEIMHAFVDTMDFTKRRFVDALRQFL
NCU07658.1	PEDTPDAIAKFLISEERLDKAQIGEYLGEGDQKNIDIMHAFVDTMDFTKKRFVDALRQFL
FG04168.1 	PSDSPKDIAEFLLREDKLDKAQIGEYLGEGEQKYIDIMHAFVDTMEFAKRRFVDSLRQFL
AN6709.1  	RSDSPEDIAAFILRNDRLDKAMIGEYLGEGDAENIATMHAFVDMMDFSKRRFVDALRSFL
          	 .* *. ** *:: :::**** ********: : *  ****** *:*:*:****:**.**

MG09877.1 	QSFRLPGEAQKIDRFMLKFANRYVMGNPNAFANADTAYVLAYSVILLNTDLHSSKIA-RR
NCU07658.1	QAFRLPGEAQKIDRFMLKFANRYMLGNPKAFANADTPYVLAYSVIMLNTDLHSSKIV-RR
FG04168.1 	QSFRLPGEAQKIDRFMLKFAERYVLGNPNAFANADTAYVLAYSVILLNTDLHSVKIA-KR
AN6709.1  	QHFRLPGEAQKIDRFMLKFSERYVTQNPNAFANADTAYVLAYSVILLNTDQHSSKMKGRR
          	* *****************::**:  **:*******.********:**** ** *: .:*

MG09877.1 	MSKEDFIKNNRGINDNADLPEEYLISIYDEIASNEIVLTSERQAAAAAGTVPPQATGIAA
NCU07658.1	MTKEDFIKNNRGINDNANLPDEYLISIYDDIANNEIVLKSEREAAAAAGTLPAQSTGLAA
FG04168.1 	MSKEEFIKNNRGINDNADLPDDYLLGIYDEIAAHEIVLKSERDAAAAAGNVPAQSTGIAA
AN6709.1  	MTKEDFIKNNRGINDNQDLPDEYLGSIFDEIANNEIVLDTEREQAANAAHPAPVPSGLAS
          	*:**:*********** :**::** .*:*:** :**** :**: ** *.  .. .:*:*:

MG09877.1 	GIGQAFS--SRDYQREAYVQQSEEISLRSEQLFKNLFKSQRRNAE-MAGIKFIPATSFKH
NCU07658.1	GLGQAFSNVGRDLQREAYAQQSEEIAIRSEQLFKDLYRSQRKNAQKMGGIKFIPATSFKH
FG04168.1 	GLGQALSNVGRDLQREAYMQQSEEIALRSEQLFKDLFKSQRR----KAGTKYILATSFKH
AN6709.1  	RAGQVFATVGRDIQGERYAQASEEMANKTEQLYRSLIRAQRKTAVKEALSRFIFATSVQH
          	  **.::. .** * * * * ***:: ::***::.* ::**:.: . .  ::* ***.:*

MG09877.1 	VGPMFDVTWMSFFSALSSQLQKALNLEVIKLCLEGMKLAIKIACFFDLPTPREAFISAVK
NCU07658.1	VSPMFDVTWMSFFSALSSQMQKTHNLDVNKLCLEGMKLATKIACFFDLSTPREAFISALK
FG04168.1 	VSPMFSVTWMSIFSTLSSQIQKSHNLEVNKLCLEGMKLATQIACLFDMSTPREAFMSALK
AN6709.1  	AGSMFNVTWMSFLSGLSAPMQDTQNLKTIKLCMEGMKLAIRISCTFDLETPRVAFVTALA
          	...**.*****::* **: :*.: **.. ***:****** :*:* **: *** **::*: 

MG09877.1 	NTANLNNPQEVFAKNLEALRVLLELGYTEGNYLRQSWKDILMCVSQLERLQLMAGGVDAN
NCU07658.1	NTANLNNPQEIQAKNVEALKVILELGQTEGNLLKESWKDVLLCISQLDRLQLISGGVDES
FG04168.1 	NTTNLNNPQEMLAKNIEALKVVLELGQTEGNVLRESWKDVLMCISQLDRLQLISGGVDES
AN6709.1  	KFTNLGNVREMVAKNVEAVKILLDVALSEGNHLKSSWRDILTCVSQLDRLQLLSDGVDEG
          	: :**.* :*: ***:**::::*::. :*** *:.**:*:* *:***:****::.*** .

MG09877.1 	SVPDVSKARFQPPARDNPSDARKSAATKRQRQRSNTATHGLNTDIAYEILSDEMIKSMDR
NCU07658.1	AVPDVSKARFVPPPRSDSTDSRKSMAAKRHRPRSNTGPQGVSMEIALESRSDEVIKSVDR
FG04168.1 	AVPDVSKARFIPPPRSETSDSRSSSSKKSTRARAGTSTKGFSTEIALESRSDDVIRSVDR
AN6709.1  	SLPDMSRAGVVPPSASDGP-RRSMQAPRRPRPKSITGPTPFRAEIAMESRSTEMVKGVDR
          	::**:*:* . **. .: .  *.  : :  * :: *..  .  :** *  * ::::.:**

MG09877.1 	IFTNTANLNGDAIGHFARALTEVSWDEIKVSGSNDQPRMYSLQKIVEISYYNMTRVRFEW
NCU07658.1	IFTNTAHLSGEAIVHFARALTEVSWDEIKVSGSNDSPRTYSLQKIVEISYYNMTRVRFEW
FG04168.1 	IFTNTANLTGESMVYFARALTEVSWDEIKVSGSNDMPRTYSLQKIVEISYYNMNRVRFEW
AN6709.1  	IFTNTANLSHEAIIDFVRALSEVSWQEIQSSGQTASPRTYSLQKLVEISYYNMTRVRIEW
          	******:*. :::  *.***:****:**: **..  ** *****:********.***:**

MG09877.1 	TTIWDVLGDHFNNVGCHINEAIVFFALDSLRQLSMRFMEIEELPGFKFQKDFLKPFEHVM
NCU07658.1	TNIWDVLGEHFNRVGCHVNAAIVFFALDSLRQLSTRFMEIEELAGFKFQKDFLKPFEHVM
FG04168.1 	SNIWEVFGEHFNRVGCHNNMNIVFFALDSLRQLSMRFMEIEELAGFKFQKDFLKPFEHVL
AN6709.1  	SKIWEVLGQHFNQVGCHSNTTVVFFALDSLRQLSMRFMEIEELPGFKFQKDFLKPFEHVM
          	:.**:*:*:***.**** *  :************ ********.***************:

MG09877.1 	SNSSNIRVKDMVLRCLIQMIQARGENIRSGWRTMFGVFTVAARDPS-~-ESIVSMAYENV
NCU07658.1	SNSNNITVKDMILRCLIQMIQAKGDKIRSGWRTMFGVFTVAAKDQY-~-ESIVNLAYEHV
FG04168.1 	ANTHNVTVKDMVLRCLIQMIQARGDNIRSGWRTMFGVFTVAAREPH-~-ESIVNLAYENV
AN6709.1  	SNSNAVTVKDMILRCLIQMIQARGDNIRSGWKTMFGVFSFAAREPYD1TEGIVNMAFEHV
          	:*:  : ****:**********:*::*****:******:.**::  . :*.**.:*:*:*

MG09877.1 	SHVYKTRFGVVISQGAFTDLIVCLTEFSKNMKFQKKSLQAMETLKSIIPRMLKAPECPLS
NCU07658.1	LHVYKTRFGVVITQGAFTDLIVCLTEFSKNMKFQKKSLQAMETLKSIIPKMLKTPECPLS
FG04168.1 	NQVYKTKFGVVISQGAFTDLIVCLTEFSKNLKFQKKSLAALELLKSLIPTMLKTPECPLS
AN6709.1  	TQIYNTRFGVVITQGAFPDLVVCLTEFSKNTRFQKKSLQAIELLKSTVAKMLRTPECPLS
          	 ::*:*:*****:****.**:********* :****** *:* *** :. **::******

MG09877.1 	RKYTSNNQLKEASTIESPIKSPGQSRTSIEEAFWFPVLFAFHDVLMTGEDLEVRSN~ALN
NCU07658.1	HKQNSEVNSAEMALNAATKPTGQETGTTVEEGFWFPVLFAFHDVLMTGEDLEVRSN~ALN
FG04168.1 	QKYN-NIPPPDGALQTSEKRS--RSNTSVEEGYWFPVLFAFHDVLMTGEDLEVRSN~ALE
AN6709.1  	HRSS-----TEAFHEDSTNLTQQLTKQSKEEQFWYPILIAFQDILMTGDDLEARSR2ALT
          	:: .      :     :   :   :  : ** :*:*:*:**:*:****:***.**. ** 

MG09877.1 	YFFEALLRYGGDFPPEFWDTLWRQQLYPIFMVLRSRPEMSNVLNHEELSVWLSTTMIQAL
NCU07658.1	YFFDALLKYGATFPLGFWDILWRQQLYPIFMVLRSKPEMSNALSHEELSVWLSTTMIQAL
FG04168.1 	YFFETLLRYGGTFPAEFWDILWRQQLYPIFMVLRSRPEMSNVLNHEELSVWLSTTMIQAL
AN6709.1  	YLFDTLIRYGGSFPQEFWDVLWRQLLYPIFVVLQSKSEMSKVPNHEELSVWLSTTMIQAL
          	*:*::*::**. **  *** **** *****:**:*:.***:. .****************

MG09877.1 	RNMITLFTHYFDALEYMLDRFLELLALCICQENDTIARIGSNCLQQLILQNVTKFTPEHW
NCU07658.1	RNMITLFTHYFNPLEYMLDRFLELLALCILQENDTIARIGSNCLQTLILQNVEKFTHEHW
FG04168.1 	RNMITLFTHYFDALEYMLDRFLELLALCICQENDTISRIGSNCLQQLILKNVTKFKPEHW
AN6709.1  	RHMITLFTHYFDALEYMLGRVLELLTLCICQENDTIARIGSNCLQQLILQNVEKFQKDHW
          	*:*********:.*****.*.****:*** ******:******** ***:** **  :**

MG09877.1 	AKIVGAFCELFERTTAYQLFSATTINSTASLSLSPPPSGLDFGGPLSPGLDSPKLDEKSL
NCU07658.1	QKIVDTFCELFDKTTAHQLFKAATISTPLS-----ASNGLDYASQLSPSADGADADTRSL
FG04168.1 	NKLVGAFCELFERTTAYQLFSATAINNTA--SISPPPNGLEFS---AGATDATPVDEKSL
AN6709.1  	NKTVGAFIELFNKTTAYELFTAATTMATVT-----------LKTPSAPTANGQLADTHDT
          	 * *.:* ***::***::**.*::   . :                :   :.   * :. 

MG09877.1 	KINGGEENGYLSDGESTVDTSAEAG----EDPLKTPTAHPSQTVVAAATDNV---PSPVL
NCU07658.1	KINGDNDAG--SETSSIHQTPHTNGGINPEEDSHSSVHNRLTASTALSSHPPNTPGGQGL
FG04168.1 	KINNRKD----SLDEDSSAIPQPDD----DDDLQTPTGDG---------------PHVAL
AN6709.1  	VQDPTESS---PAQETSTEPPKLNG----TQDTTAEHEDGDMPAAS----------NTEL
          	  :  :.    .  .     .   .     :   :   .   .  :             *

MG09877.1 	EDYKPSSHLQQQPVVVTAARRRFFNRIISRCVLQLLMIETVNELFSNEAVYAQIPSSELL
NCU07658.1	EEFKPSTTLQQQPPVVTAARRRFFNRIISRCVLQLLMIETVQELFSNDSVYSHIPSPLLL
FG04168.1 	EEFKPSSNLQQQPVVVTAARRRFFNRIISRCVLQLLMIETVNELFSNDTVYAHIPSQELL
AN6709.1  	EDYRPQSDTQQQPAAVTAARRRYFNRIITSCVLQLLMIETVHELFSNDKVYAQIPSHELL
          	*:::*.:  **** .*******:*****: ***********:*****: **::***  **

MG09877.1 	RLMGLLKKSFLFARRFNNDKELRMRLWREGFMKQPPNLLKQESGSAATYIAILFRMFADQ
NCU07658.1	KLMSLLKRSYLFAKRFNADKDLRMRLWREGFMKQPPNLLKQESGSAAVYVSILFRMFADT
FG04168.1 	RLMSLLKRSFQFARKFNEDKELRMRLWREGFMKQPPNLLKQESGSAATYISILFRMFADD
AN6709.1  	RLMGLLKKSYQFAKKFNEDKELRMQLWRQGFMKSPPNLLKQESGSAATYVHILFRMYHDE
          	:**.***:*: **::** **:***:***:****.*************.*: *****: * 

MG09877.1 	APGRRESKSDVENALVP~LCKDIIRGYISLEEESQHRNILAWRPVVVDVLEGYAA-----
NCU07658.1	SPERLASKADVERALVP~LCLDIVRVYVSLEEESQNRNIQAWRPVVVEVLNGFAGGVSNP
FG04168.1 	APERLSSRPDVEAALVP~LCKDIVHGYTTLEEESQHRNIVAWRPVVVDVLEGYTT-----
AN6709.1  	REERKSSRSETEAALIP2LCVDIISGFVRLDEDSQHRNIVAWRPVVVDVIEGYTN-----
          	   *  *:.:.* **:* ** **:  :  *:*:**:*** *******:*::*::      

MG09877.1 	----------LPESAFRNHAKAFYPLVVDLVAKELSG---ELRSALLLVLRRVGEYELGI
NCU07658.1	EKGEMKEGRGFGEEDFKKHLHEVYPVVIELLGKELGQGERELRQALLGVLRRVGEVGLGI
FG04168.1 	----------FPEDAFKKHIPDFYPLAVELLTKDLTP---DLRASLLLVLRRVGEVALGI
AN6709.1  	----------FPAEGFDKHIDTFYPLAVDLLGRELNS---EIRLAIQGLFQRIGEARLGL
          	          :  . * :*   .**:.::*: ::*     ::* ::  :::*:**  **:

MG09877.1 	EGMATSSSYNHRGHNRSDSVVTMSTEGGGGGHSPSVEVESGDASSRFMGRE1KSRNKR2R
NCU07658.1	EGLVGSS------NSNSNSTGRLSS--------------------------~------~-
FG04168.1 	EGMTHAR-------QRQDSVASRMT------------VEP-----------~------~-
AN6709.1  	PVRPTPTP-----VSPRHSVSEHPS--------------------------~------~-
          	     . .      .  .*.    :                                   

MG09877.1 	PRWPFLINSTRPATSTVGTENHQLSYYLLVVLHNPTNDECGVLGGTLEPIATLPTA2VSI
NCU07658.1	--------------------------------------------------------~---
FG04168.1 	--------------------------------------------------------~---
AN6709.1  	--------------------------------------------------------~---
          	                                                            

MG09877.1 	APCLLKATQWAADMSLFGFNPFGLGSREPAAPQTLESRTLEAVADHIKSGKVKRITVMTG
NCU07658.1	------------------------NHRRQDTLVMSGNGRMAYGHDRTES----VVSTMTV
FG04168.1 	-----------------------MGDREADARRML-------------------------
AN6709.1  	--------------------------RKHSVGRR--------------------------
          	                          *.  .                             

MG09877.1 	AGISTAAGI1PDFRSPGTGLYSNLERLKLPEPEAVFDISFFRDRPEPFYVLAKELYPGKF
NCU07658.1	GTIGGGGGG~GGYR----------------------------------------------
FG04168.1 	---------~--------------------------------------------------
AN6709.1  	---------~--------------------------------------------------
          	                                                            

MG09877.1 	QPTISHAFIALLSKKGLLQMNFTQNIDCLERQAGVPGEKVIEAHGSFATQSCIECKELFP
NCU07658.1	-----------------------------EREREREGEREGEGEGEDMAR----------
FG04168.1 	------------------------------------------------------------
AN6709.1  	------------------------------------------------------------
          	                                                            

MG09877.1 	DDEMLLHVEKEIVPRCASCNGLVKPNIVFFGEPLPRTFSEKCHLVAESDLAIIIGTSLTV
NCU07658.1	------------------------------------------------------------
FG04168.1 	------------------------------------------------------------
AN6709.1  	------------------------------------------------------------
          	                                                            

MG09877.1 	YPFAGLPELVPRGSPRLLLNKVRVGQIGTRSDDVVELGSCDAGVRKLADLLGWRDELEDL
NCU07658.1	------------------------------------------------------------
FG04168.1 	------------------------------------------------------------
AN6709.1  	------------------------------------------------------------
          	                                                            

MG09877.1 	WRSVVGDAEADRQIAAKDDEQGGYSDDSSDSDPDEDMRKLAEVVNRMNLGLETSAVDDSS
NCU07658.1	------------------------------------------------------------
FG04168.1 	------------------------------------------------------------
AN6709.1  	------------------------------------------------------------
          	                                                            

MG09877.1 	SKPGDDGVPKATMKSPDETASSDNLKPEDPSMGDKHKMEQSNSTQVRSVESQQKKGTGAS
NCU07658.1	------------------------------------------------------------
FG04168.1 	------------------------------------------------------------
AN6709.1  	------------------------------------------------------------
          	                                                            

MG09877.1 	NQVEEAVSTHGSAGEAKTKNTRPDTSQQAQPPVNALAAPVQSSSGGKVEE0A0LTVSTQQ
NCU07658.1	--------------------------------------------------~-~-------
FG04168.1 	--------------------------------------------------~-~-------
AN6709.1  	--------------------------------------------------~-~-------
          	                                                            

MG09877.1 	DFSQTREPPAVWVLQVK0KLNLEGWDRGIGHYLLQRLSELPRDAPKPHVVTSSGGNAGLA
NCU07658.1	-----------------~------------------------------------------
FG04168.1 	-----------------~------------------------------------------
AN6709.1  	-----------------~------------------------------------------
          	                                                            

MG09877.1 	AVHAARNVGLPCTVVVPLLIKPLMIEKLKAAGAHEVVRHGATWQETDDYVRHEFMAKSEV
NCU07658.1	------------------------------------------------------------
FG04168.1 	------------------------------------------------------------
AN6709.1  	------------------------------------------------------------
          	                                                            

MG09877.1 	PTIYIPPFNHPDVWTGHSTMVHEMAKQMPTGTLPDAIICSVGGGGLLNGICQGVDDVAAA
NCU07658.1	------------------------------------------------------------
FG04168.1 	------------------------------------------------------------
AN6709.1  	------------------------------------------------------------
          	                                                            

MG09877.1 	ADPSRAKPIILAVETEGADSLNASVKAGELVELPGITSLATSLGAKKVTVKTLEYAQQDH
NCU07658.1	------------------------------------------------------------
FG04168.1 	------------------------------------------------------------
AN6709.1  	------------------------------------------------------------
          	                                                            

MG09877.1 	VRSHVLTDAEAAMGCWKLADDERLLVELACGVSVSLCYGGRLEKALGRKVKADDSVVIVL
NCU07658.1	------------------------------------------------------------
FG04168.1 	------------------------------------------------------------
AN6709.1  	------------------------------------------------------------
          	                                                            

MG09877.1 	CGGSNITVEMLREYREMFGGHAAVAEHQTSSSI
NCU07658.1	---------------------------------
FG04168.1 	---------------------------------
AN6709.1  	---------------------------------
          	
```
